# Chloroforming Mice

**Published:** 1882-09

**Authors:** 


### CHLOROFORMING MICE.

A French Surgeon says, that on chloroforming some mice and lifting them by their tails, they tried to bite, but on laying them again in a horizontal position, they resumed insensibility. Acting on this hint, when a patient showed signs of collapse under a dose of chloroform, he dropped the patient's head over the bedside and raised the feet quite high. The patient at once became conscious; when laid straight on the bed he became insensible again, and a return to lowering the head and raising the feet for ten minutes was required to fully counteract the chloroform. It is thought, that by aid of this treatment, anæsthetics may be used with a high degree of safety.

